# Targeting Alcohol Septal Ablation in Patients with Obstructive Hypertrophic Cardiomyopathy Candidates for Surgical Myectomy: Added Value of Three-Dimensional Intracoronary Myocardial Contrast Echocardiography

**DOI:** 10.3390/jcm10102166

**Published:** 2021-05-17

**Authors:** Giovanni La Canna, Iside Scarfò, Irina Arendar, Antonio Colombo, Lucia Torracca, Davide Margonato, Matteo Montorfano, Ottavio Alfieri

**Affiliations:** 1 Applied Diagnostic Echocardiography Unit, IRCCS Humanitas Clinical and Research Center, 20089 Rozzano, Italy; isidestella.scarfo@gmail.com (I.S.); arendaririna@hotmail.com (I.A.); 2 Interventional Cardiology Unit, IRCCS Humanitas Clinical and Research Center, 20089 Rozzano, Italy; antonio.colombo@hunimed.eu; 3 Cardiac Surgery, IRCCS Humanitas Clinical and Research Center, 20089 Rozzano, Italy; lucia.torracca@humanitas.it; 4 Interventional Cardiology Unit, IRCCS San Raffaele Scientific Institute, 20132 Milan, Italy; dvdmrgnt@gmail.com (D.M.); matteo.montorfano@hsr.it (M.M.); 5 Cardiac Surgery Unit, IRCCS San Raffaele Scientific Institute, 20132 Milan, Italy; ottavio.alfieri@hsr.it

**Keywords:** hypertrophic cardiomyopathy, left ventricular obstruction, alcohol septal ablation, surgical myectomy, myocardial contrast echocardiography, three-dimensional echocardiography

## Abstract

Background: Myocardial contrast two-dimensional echocardiography (MC-2DE) is widely used to address alcohol septal ablation (ASA) in obstructive hypertrophic cardiomyopathy (HCM). Owing to its limited cut-planes, MC-2DE may inaccurately identify the contrast misplacement associated with an unsuccessful or complicated ASA outcome. Objective: The aim of this study was to assess the added value of myocardial contrast three-dimensional echocardiography (MC-3DE) compared with MC-2DE to identify the appropriate matching between the target septal zone (TSZ) and coronary artery branch for safe and long-term effective ASA in HCM patients. Methods: A consecutive series of 52 symptomatic obstructive HCM patients referred for isolated surgical myectomy (SM) was analyzed with MC-2DE and MC-3DE following injection of echocontrast into one or more septal branches. MC-2DE and MC-3DE patterns were categorized according to complete (*Type 1*) or incomplete (*Type 2*) TSZ covering, high-risk (*Type 3*) exceeding TSZ, or life-threatening outside TSZ distribution (*Type 4*). Results: MC-2DE per patient analysis showed a *Type 1 pattern* in 32 patients and *Types 2–4* in the remaining 20 patients; subsequent MC-3DE analysis provided a re-phenotyping of MC-2DE findings in 22 of the 52 patients (42%), showing a high-risk *Type 2 pattern* in 17 of the 32 patients with *Type 1*, and a new life-threatening *Type 4* in three patients with *Type 2,* respectively. All patients with MC-3DE *Type 1 pattern* underwent safe and effective ASA with a long-term uneventful follow-up, while the remaining patients underwent SM. Conclusions: Refining high risk or life-threatening contrast misplacement, MC-3DE is more accurate than conventional MC-2DE to target safe and long-term effective septal reduction with ASA in obstructive HCM patients referred for isolated SM.

## 1. Introduction

Alcohol septal ablation (ASA) is regarded as an effective alternative to surgical myectomy (SM) to treat left ventricular (LV) obstruction in patients with symptomatic hypertrophic cardiomyopathy (HCM) [1,2,3,4,5,6,7]. Successful ASA requires alcohol injection into the coronary branch supplying the septal zone involved in the LV obstruction, the so-called target septal zone (TSZ), to induce an appropriate scar mimicking LV outflow tract SM-reshaping. Identification of the coronary artery branch supplying the TSZ is crucial to achieve effective ASA without complications, including intraprocedural events (due to alcohol distribution outside the TSZ) or late arrhythmogenic burden (due to excessive myocardial scarring). Even though several nonrandomized studies advocate a safe and effective outcome comparable to SM, in the clinical scenario, ASA therapy is commonly reserved to the elderly or patients with comorbidity-related high surgical risk. Recently, due to greater expertise and patient preference, ASA has also been extended to HCM patients with low-risk SM [8,9,10]. However, without head-to-head studies, the feasibility of ASA in an HCM population initially selected for SM remains unexplored, which challenges the effectiveness of ASA in comparison with SM. Intracoronary myocardial contrast echocardiography (MCE) is widely used to select patients suitable for ASA [11,12,13]. However, MCE, when performed with two-dimensional echocardiography (MC-2DE) may be affected by the difficulty to quickly assess overall myocardial contrast distribution following septal coronary branch injection using multiple images in succession. We can hypothesize that 3D analysis may enhance the accuracy of MCE studies, mapping a complete myocardial distribution of selected coronary artery branches for subsequent alcohol injection and improving the criteria for ASA suitability. Even though its advantages exceed the limitations of 2D imaging, the experience of MCE with three-dimensional echocardiography (MC-3DE) to select appropriate candidates for ASA vs. SM is still scarce [13,14,15]. The aim of this study was to assess the added diagnostic value of MC-3DE compared with conventional MC-2DE to identify septal anatomy-coronary artery branch matching for safe and effective ASA in a consecutive series of symptomatic HCM patients initially referred for SM treatment of LV obstruction.

## 2. Materials and Methods

### 2.1. Study Population

We retrospectively analyzed 171 consecutive patients in our database from 2004 to 2010 with HCM referred for surgical treatment of LV obstruction because symptoms were unresponsive to optimal medical therapy. The setting was a tertiary referral center with expertise in septal reduction therapy, including both surgical and alcoholic septal ablation. Each patient fulfilled HCM diagnostic criteria, including an unexplained hypertrophy (maximal wall thickness >15 mm) in the absence of other cardiac or systemic conditions capable of producing a similar degree of LV hypertrophy [16]. We excluded 119 patients from the study group based on the following criteria: septal thickness in site of target zone <18 mm (31 patients); isolated mid LV obstruction due to septal contact of papillary muscle of mitral valve (10 patients); isolated or associated left ventricular apical obstruction or aneurysm (3 patients); concomitant aortic valve disease (20 patients); structural mitral valve abnormalities requiring concomitant surgical procedures (30 patients); significant coronary artery disease (25 patients). The calculated sample size was 32 patients, using a two-side significance level alfa of 0.05% and a power of 0.8, assuming as clinically relevant a minimum of 30% difference between 2D and 3D diagnostic outcome on ASA feasibility.

We enrolled a final group of 52 patients that fulfilled HCM diagnostic criteria with at-rest or inducible SAM-related LV obstruction (intraventricular gradient > 50 mm Hg) and septal phenotype suitable for isolated surgical reduction therapy by SM, prospectively undergoing MC-2DE and MC-3DE using a first-generation contrast agent. The clinical and echocardiographic characteristics are reported in Table 1.

### 2.2. Echocardiographic Study

Following clinical examination, all patients underwent standard transthoracic echocardiography (TTE) at rest in the morning under fasting conditions using an iE33 Philips ultrasound model connected to a probe capable of 2D and 3D imaging (Philips Ultrasound, Andover, MA, USA). Medical treatment was maintained in all patients. Standard TTE was performed during held end-expiration to evaluate, in addition to conventional parameters, the septal thickness and the presence and mechanism of LV obstruction. Septal measurement was made with a long axis parasternal view using 2D-guide M-Mode imaging to optimize the perpendicular alignment of the ultrasound beam with the septal structure. Careful attention was paid to identify aberrant para-septal bundles as a source of overestimation of septal thickness. Measurement of intraventricular gradient (IVG) was performed with continuous-wave Doppler interrogation of the velocity signal from an apical window, and calculated using the modified Bernoulli equation (i.e., gradient = 4v2 where v = peak left ventricular outflow tract velocity). Particular attention was paid to optimize alignment with left ventricular outflow tract flow for accurate IVG measurement, avoiding mistaken estimation based on the concomitant mitral regurgitant jet sample. As previously described [17], the LV obstruction mechanism is classified as SAM-related when it is due to septal contact of systolic anterior motion of mitral valve (SAM), non-SAM related when it is due to mid-cavity septal contact of papillary muscle, abnormal papillary insertion in the LVOT or apical obstruction, or combined (SAM-related and non-SAM-related). Concomitant SAM related mitral regurgitation was graded using vena contracta measurement with a diameter >7 mm as cut-off severity. Following at-rest evaluation, the patients underwent TTE during Valsalva maneuver. In patients without significant IVG, a semisupine cycloergometer exercise test with 10 watt/min load incrementation every minute and a rest retest postprandial echocardiography were carried out to assess LV obstruction inducibility [17].

### 2.3. Myocardial Contrast Echocardiography 

To identify appropriate coronary artery/TSZ matching, MCE was carried out with the injection of a contrast agent into at least one septal coronary artery branch. Following conventional coronary angiography, a temporary pacemaker was positioned in patients without a permanent pacemaker or cardioverter-defibrillator (ICD) in place. The estimated TSZ-related septal coronary artery was cannulated with a standard percutaneous coronary intervention wire. Following balloon insufflation, angiographic contrast was injected to check the complete balloon occlusion to avoid a backflow of the contrast agent into the anterior descending coronary vessel. Adjusting 2° harmonic setting for image acquisition and low mechanical index less than 1 to determine bubble destruction, the echo-contrast agent (300 mg/mL Levovist^®^ a galactose-based contrast agent; Bayer Schering Pharma, Berlin, Germany) was injected through the central lumen of the occluded balloon catheter during 2D-TTE and full volume 3D-TTE monitoring. Additional septal coronary branches were injected to attempt the best matching of septal branches with TSZ.

Two-dimensional echocardiography with standard and off-axis apical views were used to image left and right ventricular segments. Following intracoronary contrast injection in the selected coronary branch, particular attention was paid to obtain clear visualization of contrast appearance inside and outside the TSZ. Subsequent 3D-TTE was quickly acquired using the apical view with a single-beat full volume mode maintaining a low mechanical setting less than 1 and adjusting the scansion width to obtain comprehensive left and right ventricular-cavity images with maximum possible frame rate acquisition (at least 30 Hz). Both 2D-TTE and 3D-TTE images were acquired during contrast injection, using a 20-consecutive-beat-cycle-length recording for subsequent cine-loop review.

### 2.4. Myocardial Contrast Echocardiography Analysis

Echocardiographic images were acquired and stored in digital format for the subsequent analysis of the “contrast effect” distribution following septal coronary artery injection. The contrast effect was visually assessed by two observers as an increase of myocardial echogenicity using as a reference the pericardium signal without changing baseline acoustic gain. Following 2D-TTE analysis, the acquired full volume 3D-TTE images were immediately analyzed with a multiple cut plane (at least 6 slices from base to apex of the heart) using the standardized segment model nomenclature. The MC-2DE and MC-3DE myocardial contrast were categorized using 4 patterns according to contrast distribution: complete covering of TSZ (*Pattern 1*); incomplete covering of TSZ (*Pattern 2*); high-risk pattern (outside the TSZ, involvement of right site of interventricular septum, longitudinal extension beyond 1/3 of interventricular septum) (*Pattern 3*); life-threatening pattern (right or left ventricle free wall, papillary muscle) (*Pattern 4*). Lacking identification of an adequate coronary artery septal branch supplying the TSZ, each patient was classified using the worst observed pattern.

The patients with Pattern 1 were considered suitable for safe and effective ASA (*Group A*), while the patients with patterns 2–3-4 were defined unsuitable for ASA (*Group B*).

### 2.5. Alcohol Septal Ablation

Following careful and detailed information with the patient about the advantages and disadvantages of both therapies, every patient in Group A accepted septal reduction with ASA rather than SM and provided written informed consent. All patients of Group B underwent SM guided by intraoperative transesophageal and open chest-epicardial echocardiography within seven days of MCE study.

Based on MC-3DE results, to attempt septal reduction a small amount of ethanol (max 1–2 mL) was slowly injected into the coronary artery septal branch supplying the TSZ through the central lumen of the balloon catheter under fluoroscopic, hemodynamic, and electrocardiographic monitoring. During the ASA procedure TTE monitoring aimed to evaluate the overlap of alcohol distribution within TSZ, according to previous myocardial contrast findings, and follow the impact on LV obstruction and mitral regurgitation. Following the final alcohol injection, the balloon catheter was withdrawn, and a subsequent angiogram was performed to detect a complete occlusion of the septal branch with normal flow in the left anterior descending artery. Measurements of the IVG at rest, during post-extrasystolic ventricular beat, and following pharmacological stress with i.v. isoproterenol were carried out. Only one septal branch was performed to await the hemodynamic effect of the ultimate LVOT-reshaping after induction of the ethanolic scar. A short-time (48 to 72 h) clinical, electrocardiographic and echocardiographic surveillance was carried out at the intensive care unit, as well as cardiac enzymes every 6–8 h.

### 2.6. Follow-Up

The post-ASA clinical course was followed either in our outpatient clinic or by the referring cardiologists. Clinical examination and TTE studies were performed at rest under fasting and eating conditions. The symptomatic patients without at rest LV obstruction also underwent exercise echocardiography to exclude inducible LV obstruction. Clinical status, medical treatment or need to change medication, arrhythmias, death, and pacemaker or ICD implantation were considered as events. In patients with an implanted pacemaker or ICD, the device’s memory and function were assessed, and ICD discharge was annotated.

### 2.7. Statistical Analysis

Continuous variables were expressed as mean ± SD when normally distributed, median (IQR) when non-normally distributed, and qualitative variables as a percentage. Paired t tests and repeated measures ANOVA or Friedman Test when appropriate were used to compare pre- and post-procedure variables within patients. The Bonferroni correction was used for post hoc analysis of significant results. Unpaired comparisons were performed using Student’s t test or Wilcoxon’s rank sum test (Mann-Whitney U test) as appropriate according to the distribution. Proportions were compared with the chi-square test or Fisher’s exact test. The inter- and intra-observer agreement in the echocardiographic images was assessed using the K test. Phenotype categorization between MC-2DE and MC-3DE was compared using McNemar test. *P* values < 0.05 were considered statistically significant.

## 3. Results

**Figure 1:**
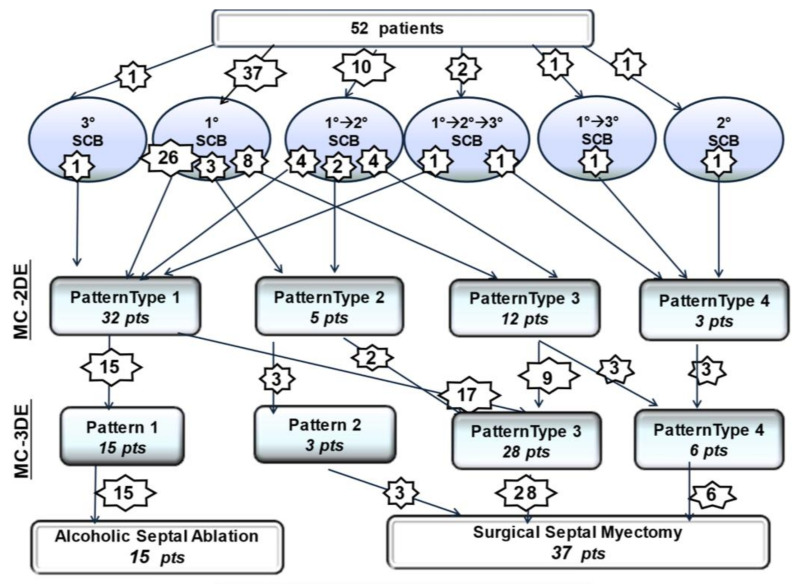
Intracoronary myocardial contrast echocardiography phenotyping in 52 patients with HCM. Star: number of patients; **SCB**: septal coronary branch; Pattern Type 1–3 (refer to text); **MC-2DE**: myocardial contrast two-dimensional echocardiography; **MC-3DE**: myocardial contrast three-dimensional echocardiography.

The outcome of intracoronary MC-2DE and MC-3DE in the 52 HCM patients is reported in Figure 1. A complete intra- and interobserver agreement (100%, K 1.00) was achieved in the classification of the pattern type of myocardial contrast effect following the injection into the coronary artery branches.

Fifty of the 52 patients underwent isolated or combined cannulation of the first septal branch, while the remaining two patients, owing to hypoplasia of the first septal branch, underwent isolated cannulation of the second or the third septal branch, respectively. In two patients, the injection of a contrast agent was performed in both sub-branches of the duplicated septal branch.

### 3.1. Two-Dimensional Myocardial Contrast Echocardiography Phenotypes

In 37 of the 52 patients undergoing echo-contrast injection into the first septal coronary branch alone, MC-2DE analysis showed a *Type 1* pattern in 26 patients, *Type 2* in 3 patients, and *Type 3* in 8 patients. In 13 of the 52 patients without a *Type 1* pattern following the first septal coronary analysis, the subsequent contrast injection into the subselective first septal branch or into the second and/or third septal branches showed a *Type 1* pattern in five patients, *Type 2* in two patients, *Type 3* in four patients, and *Type 4* in two patients. In the last two of the 52 patients, owing to first coronary septal branch hypoplasia, the isolated cannulation of the second and third septal branches showed a *Type 1* pattern and *Type 4* pattern, respectively. A patient with a duplicated first septal branch showed a different perfusion pattern (*Type 4* high-risk for the first sub-branch and *Type 1* for the second sub-branch). The first septal branch matched the TSZ in 26 of the 52 (50%) patients, while in the remaining patients there was TSZ mismatch. Of the 63 cannulated septal branches, 32 (51%) showed appropriate TSZ matching, while the remaining 31(49%) revealed TSZ mismatch. Based on per-patient MC-2DE analysis, 32 of the 52 (61%) could have been suitable for ASA, using the first septal coronary artery branch in 26 patients, while using the second or third septal branch in six patients, respectively.

### 3.2. Three-Dimensional Myocardial Contrast Echocardiography Phenotypes 

**Figure 2:**
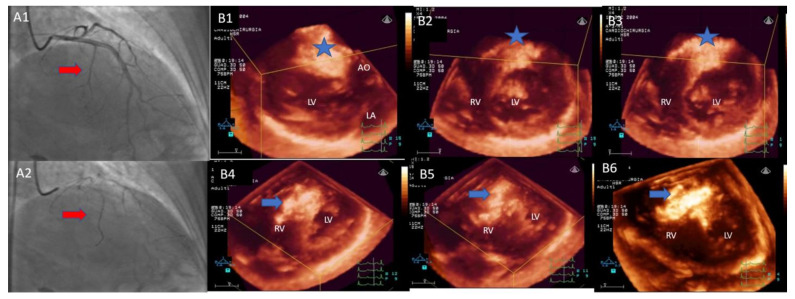
Three-dimensional images of intracoronary myocardial contrast echocardiography. Following contrast agent injection into the first septal branch (**A1**,**A2**, red arrow), three-dimensional multiple slice reconstruction from base to cardiac apex (**B1**–**B6**) shows myocardial contrast effect in the basal and mid anterior septum (blue star) extending to the moderator band and a large area of the free-wall of the right ventricle (blue arrow). Owing to the high risk of life-threatening extended septal myocardial infarction involving the right ventricle, the patient was considered unsuitable for ASA. **AO**: aorta; **LA**: left atrium; **LV**: left ventricle; **RV**: right ventricle; **ASA**: alcohol septal ablation.

Subsequent MCE-3DE analysis provided a re-phenotyping of myocardial perfusion patterns in 22 of the 52 patients (42%). Seventeen of the 32 patients with *Type 1* pattern MC-2DE showed a high-risk pattern (*Type 3*), including contrast effect extension to the right boundary of the interventricular septum (6), longitudinal extension beyond 1/3 of the interventricular septum (6), contrast effect outside *TSZ* (5). A life-threatening pattern (*Type 4*) was confirmed in three patients and observed in three patients with *Type 3* pattern MC-2DE, including a contrast effect distribution involving the moderator band and the free wall of the right ventricle (2), the left ventricular free wall (2), and the papillary muscle (2). Figure 1 reports a detailed change of perfusion pattern observed following MC-3DE analysis. We observed inconsistency between MC-2DE and MC-3DE in the categorization of the TSZ-coronary artery branch matching with significantly greater accuracy of MC-3DE compared with MC-2DE (65% vs. 29%, respectively; *p* < 0.001) for the identification of high-risk or life-threatening patterns. Based on per-patient MC-3DE analysis, we found that only 15 of the 52 (29%) patients referred for isolated SM showed an appropriate septal-coronary phenotype for safe and effective ASA applicability. Figure 2 reports a patient with life-threatening *Type 4* pattern MC-3DE.

**Figure 3:**
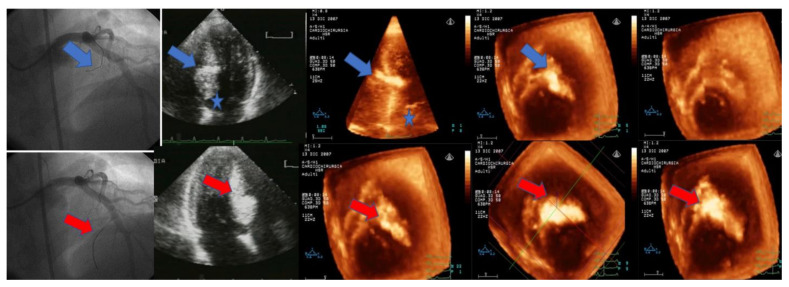
Echocardiographic images of intracoronary MCE in a patient with HCM. Following echo-contrast agent injection into the first septal coronary branch, MC-DE shows a contrast effect in the mid-interventricular septum outside TSZ, confirmed by subsequent MC-3E analysis with multiple slice reconstruction from base to cardiac apex (Type 3 pattern); following subsequent echo-contrast agent injection into the second septal coronary branch, MC-2DE images reveals a large septal opacification, which is confirmed by MC-3DE multiple reconstruction showing myocardial contrast effect extension along the overall interventricular septum (*Type 3 pattern*). Owing to the high risk of extended septal myocardial infarction, the patient was considered unsuitable for ASA.

Figure 3 shows MCE phenotyping in a patient with a HCM following sequential injection of echo-contrast agent into the first and second septal branches, respectively.

### 3.3. Alcohol Septal Ablation

**Figure 4:**
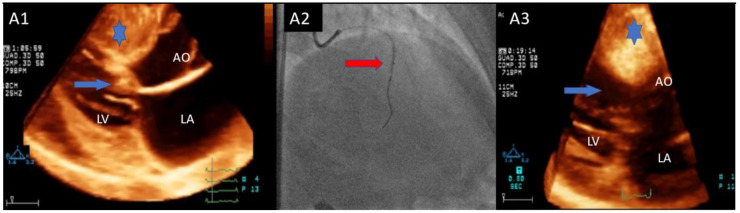

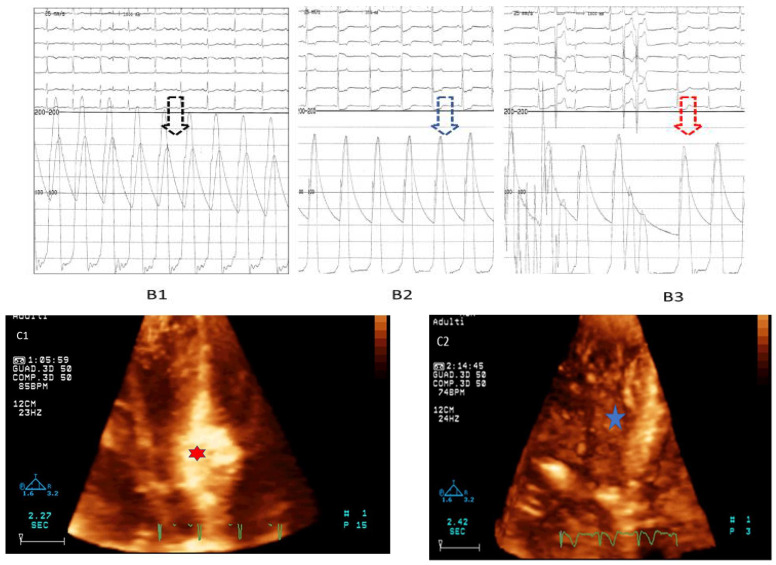
Successful alcohol septal ablation. (**A1**): 3D-TTE showing the septal contact of systolic anterior motion of mitral valve (blue arrow) (target septal zone: blue star); (**A2**): Cannulation of the first coronary septal branch (red arrow) with subsequent opacification of target septal zone (blue star) at MC-3DE following intracoronary echo-contrast injection (**A3**); (**B1**–**B3**): Intraprocedural hemodynamic monitoring during alcohol injection into the first coronary septal branch with disappearance of baseline intraventricular gradient ((**B1**), dashed black arrow) following the last alcohol injection ((**B2**), dashed blue arrow) without inducibility during the post-extrasystolic ventricular beat ((**B3**), dashed red arrow); (**C1**): 3D-TTE showing target septal zone opacification (red star) following alcohol injection; (**C2**): 3D-TTE at one-year follow-up showing optimal remodeling of target septal zone (blue star) without dynamic obstruction. 3D-TTE: three-dimensional transthoracic echocardiography; MCE: myocardial contrast three-dimensional transthoracic echocardiography.

All 15 patients of *Group A* underwent successful ASA. We found no significant differences in echocardiographic characteristics between *Group A* and *Group B*, including TSZ hypertrophy (20 ± 5 mm vs. 20 ± 5mm, NS), SAM-aortic valve distance (24 ± 3 mm vs. 23 ± 4 mm, NS), and IVG (72 ± 44 mm vs. 68 ± 43 mm, NS). During alcohol injection into the septal coronary branch, MC-3DE showed complete overlapping of myocardial opacification of alcohol distribution and the preprocedural echo-contrast area in 14 of 15 patients of *Group A*; in the remaining patient, due to a moderate extension outside the TSZ, the amount of alcohol injected was reduced. All patients showed a significant relief of IVG and SAM-related mitral regurgitation without re-inducibility during post-extrasystolic ventricular beat and following pharmacological stress with i.v. isoproterenol. No patient showed post-ASA complications, including new bundle branch block or atrioventricular block. Figure 4 Shows a patient with IVG gradient disappearance and effective long-term LV outflow tract remodeling following alcohol injection into the first septal branch.

### 3.4. Follow-Up Results

Follow-up data were available for an average of 11.6 ± 3 years (range 1–15, median 12). One 75-year-old patient died within one year due to noncardiac causes. At the longest follow-up, the remaining 14 of the 15 patients undergoing ASA were asymptomatic without significant LV obstruction in both fasting and eating at rest conditions. SAM-related LV obstruction induced by exercise test was observed in one patient. Table 2 summarizes short and long-term outcome following ASA. All patients showed a significant reduction of IVG, TSZ thickness and SAM-related MR, and a significant increase of LVOT area. The majority of the patients continued with long-term beta-blocking therapy at a lower dose than the previous preoperative regimen. In particular, we did not observe any complex ventricular arrythmias at regular 6-month 24 h-ECG Holter monitoring. A pacemaker implantation due to complete atrioventricular block was required in one patient 15 years after ASA procedure. One patient underwent ICD implantation one year after ASA based on the judgement of the referring cardiologist despite the absence of major ventricular arrhythmias.

## 4. Discussion

The main finding of this study is that MC-3DE is more accurate than conventional MC-2DE to identify optimal matching between TSZ and the supplying septal coronary artery branch, enhancing the selection of the suitable septal phenotype for safe and long-term effective ASA in HCM patients. Owing to the high prevalence of unfavorable or high-risk phenotypes, ASA may be considered not as extensively applicable as SM for the treatment of LV obstruction in the HCM population. 

Dynamic LV obstruction is associated with an unfavorable clinical course and the risk of sudden death and should be considered a therapeutic target in HCM patients [18,19,20,21]. In symptomatic patients who are unresponsive to medical therapy, septal reduction, including SM or ASA, may be clinically beneficial [1,2,3,4,5,6,7,22,23,24]. To optimize the septal reduction treatment of obstructive HCM, a careful work-up should address the obstruction mechanisms, exclude the primary causes of mitral regurgitation, and provide an accurate measurement of the thickness of the septum in the TSZ responsible for LV obstruction [25,26,27]. Dynamic LV obstruction may subtend varying myocardial hypertrophy phenotype and different mechanisms, including systolic septum contact with SAM or with the lateral papillary muscle, apical obliteration, or direct papillary muscle insertion into the LVOT. The main objective of SM is to treat LV obstruction, eliminating septal contact with SAM or/and the lateral papillary muscle. A septal thickness of at least 18 mm in the TSZ is accepted as the anatomic threshold at which SM can be performed using the conventional transaortic approach without complications, even though experienced surgeons propose septal reduction also in those patients with lower septal thickness [28,29,30]. Intraoperative echocardiography plays an important role in determining the transmural and longitudinal extension of transaortic SM, which is based on the septal thickness and the distance from the aortic valve plane of the septal contact with SAM or the papillary muscle, respectively [25,26,27,31,32,33]. Selected HCM patients with ineffective transaortic SM at intraoperative echocardiography may require additional surgical procedures, including further septal reduction (either with the transaortic or transapical approach) or mitral valve rescue surgery [31,32,33,34,35,36,37]. In patients with primary valve abnormalities or minimal septal hypertrophy in the target zone, mitral valve repair or replacement should be carried out. Depending on patient selection, the skill of the surgical team, and the high volume of performed procedures, SM is associated with low operative mortality and excellent clinical outcome, including symptom reduction and life expectancy improvement [38,39,40,41,42]. Tailoring functional anatomy subtending LV obstruction, SM should be considered as the standard procedure for the nonpharmacological treatment of LV obstruction and may also be proposed as “early therapy” to restore normal life expectancy in HCM patients [43,44,45].

Data accumulated over 20 years support the role of ASA to relief LV obstruction in HCM patients who are anatomically suitable for transaortic SM, taking advantage of the percutaneous approach involving short hospital stay, without sternotomy and extracorporeal circulation [5,46,47,48,49]. ASA consists of the alcohol injection into the coronary septal branch that supplies the TSZ, inducing myocardial damage and mimicking the results of isolated SM. The appropriate matching between the coronary septal branch and TSZ responsible for SAM-related LV obstruction is crucial to predict the therapeutic efficacy of alcohol-induced damage [10,50,51]. Unlike immediate SM results, the time-course of ASA on LV obstruction may subtend a biphasic mechanism. In the short-term after ASA, we may observe opposing effects on LV obstruction, including IVG reduction due to post-ischemic loss of contraction or IVG increase due to subsequent edema of the damaged septum, while we can expect IVG reduction in the long-term through LVOT remodeling as a result of septal scarring [52,53]. Consequently, long-term observation (at least six months) may be required to evaluate ultimate ASA effectiveness on LV obstruction. In patients referred for septal reduction with ASA, careful targeting of the correct area of the septum will maximize the effects on LV obstruction, while avoiding necrosis-related complications both inside and outside the culprit zone [54]. Common periprocedural complications of ASA include right-bundle-branch block and atrioventricular block, requiring pacemaker implantation in around 10% (range 0–40%) of patients [4,54,55,56,57]. ASA can also lead to live-threatening necrosis involving the papillary muscle, LV free wall, or right ventricle [54]. Although there are no consistent observational data, long-term complications might include threatening ventricular arrhythmias due to extensive septal scarring [58,59,60,61,62]. A varying scar site and extension following ASA were reported by CMR studies, including scarring outside the TSZ (exceeding the proximal anterior septum or extending to the right side of the septum) [54], which can limit the effectiveness of the procedure or lead to right-bundle-branch block requiring pacemaker implantation, especially in patients with preprocedural left bundle branch block. Post-ASA residual LV obstruction is associated with a higher death rate and symptom persistence requiring repeated septal reduction therapy [46,62]. Although the overall survival of propensity-matched patients is similar, ASA is associated with a higher re-intervention (35% vs. 1%) due to persistent IVG, and higher pacemaker implantation rate than in SM [63]. Even though repeated septal reduction therapy after ASA was not associated with a higher risk of major cardiovascular events over a long-term follow-up, a significant percentage of the patients enrolled in the EURO-ASA required pacemaker implantation [64]. The periprocedural complication rate can be reduced by targeting the myocardial area and by reducing the amount of alcohol injected. In the absence of randomized studies comparing ASA with SM, potential scarring burden, beyond its favorable impact on LV obstruction, should be considered when selecting a tailored procedure for each patient, seeing as minimizing the scar extension could reduce the long-term risks of ASA. Identification of the LV obstruction site and mechanism (SAM-related or SAM-free) is crucial to delineate the TSZ and septal remodeling required for successful ASA or SM. The use of myocardial contrast echocardiography has significantly improved the selection of ASA candidates, identifying the coronary septal branch supplying TSZ and predicting the alcohol-induced myocardial damage required for LV obstruction relief, without short-term complications [50,65,66,67]. MC-2DE is the most common approach to plan effective and safe ASA. However, MC-2DE requires multiple serial views to attempt complete visualization of the echo-contrast distribution pattern predicting myocardial damage following subsequent alcohol injection. Due to a limited cut-planes, MC-2DE may mislead the high-risk patterns associated with ASA peri-procedural complications or long-term arrhythmic burden. Despite the advantages of the three-dimensional approach, only small case series have been published [13,14,15] to guide the ASA procedure. In the present study, MC-3DE showed a more accurate contrast visualization than MC-2DE, providing a reliable prediction of safe and effective ASA. We observed inconsistency between MC-2DE and MC-3DE in the identification of optimal TSZ-coronary artery branch matching. Particularly, MC-3DE was able to identify high-risk or life-threatening patterns over MC-2DE. Based on per-patient MC-3DE analysis, we found that only 15 of the 52 (29%) patients referred for isolated SM showed an optimal septal-coronary phenotype for safe and effective ASA applicability. In this highly selected HCM group, ASA was associated with clinical improvement and the persistent relief of LV obstruction without repeated septal reduction therapy and in the absence of events at long-term follow-up.

The prevalence of ASA procedures vs. SM is highly variable according to center experience and patient preference [68,69]; however, no studies have systematically addressed the feasibility of ASA in a cohort of patients referred for SM. In particular, in clinical practice ASA is preferred to SM mainly in the elderly at high operative SM risk, who may have a different septal phenotype from non-elderly HCM patients at low operative SM risk. Indeed, non-elderly HCM patients at low SM risk may have a high-penetrance phenotype that is substantially different from age-dependent remodeled septal phenotype [8,10,70,71,72,73]. Thus, in the general population of HCM patients, the prevalence of ASA feasibility may differ. Accordingly, in our study ASA was successfully performed in only 15 of 52 patients (29%), while SM was carried out in the remaining 37 patients (71%). This finding is important when selecting HCM patients undergoing ASA with an expected favorable long-term clinical course. Several meta-analyses report a comparable outcome between SM and ASA [3,6,7,47]. However, the majority of the studies focus ASA results in high-risk elderly HCM population that may be affected by comorbidities, limiting the clinically beneficial impact of the procedure and long-term arrhythmic burden assessment. Recently, ASA application was extended to mildly symptomatic patients or young subjects [10,72], including HCM patients with important septal hypertrophy requiring large scarring to cover the TSZ responsible for LV obstruction [8]. Even though ASA and SM were proposed as similarly effective procedures, low feasibility ASA prevalence may be an important determinant of the effectiveness of ASA in HCM patients in alternative to SM. In addition, when performed in a highly experienced center, SM provides complete relief of LV obstruction, without any potentially arrhythmogenic septal scarring. On the other hand, unbalanced center experience and patient preference may promote a more liberal use of ASA than SM, with a significant percentage of persistent IVG requiring additional septal reduction procedures. In our study, the systematic use of MC-3DE showed low ASA feasibility in a consecutive series of HCM patients, initially proposed for isolated SM, in a center with a balanced experience in both ASA and SM. Furthermore, the patients were selected by an applied heart team, involved in HCM work-up including myocardial contrast echocardiography for ASA suitability and intraoperative echocardiography for SM strategy and monitoring. The results of this study underline that low feasibility should be considered a main determinant limiting ASA effectiveness in comparison with SM for the treatment of SAM-related LV obstruction. The selection of appropriate candidates based on MC-3DE refinement may be crucial for the clinician and patient to discuss and choose the best HCM treatment in candidates for septal reduction therapy. In this context, CMR may be useful to guide the selection of an appropriate septal reduction therapy to avoid the potential risk of additional ethanolic myocardial damage in patients with extended preprocedural LV myocardial fibrosis and provide the ultimate arrhythmogenic substrate balance in patients undergoing ASA.

### Limitation of the Study

The main limitation of the study is the small population enrolled impacting the applicability of the results in the general HCM population. Indeed, a large population may be required to establish ASA feasibility in the context of septal coronary branch variability, together with heterogeneous hypertrophy and left ventricular obstruction mechanism. Even though previously published large-population studies report a high rate (up to 90%) of ASA feasibility using 2D contrast echocardiography, a significant subject proportion required repeated ASA septal reduction or periprocedural pacemaker implantation. The use of 3D analysis could have improved the identification of patients at risk of ineffective or complicated ASA. Although this study is retrospective, the small series of HCM patients enrolled was prospectively collected, using a diagnostic work-up to identify through MCE the septal phenotype suitable for ASA as an alternative to surgical reduction. The subsequent decision to perform ASA was based on the predefined criteria of septum-coronary phenotype by the heart team and following a discussion with the patient regarding the therapeutic choice after potential MCE patterns. No patient underwent SM that was inconsistent with the MC-3DE pattern suggesting ASA feasibility. Patient enrollment was limited to the period of availability of the echo-contrast agent Levovist to reduce any additional issues related to the use of different contrast agents. Even though the study population is small, the long-term follow-up supports a favorable clinical course without the need to repeat the intervention in an appropriately selected septal phenotype.

## 5. Conclusions

In HCM patients with LV obstruction, MC-3DE was more accurate than MC-2DE in identifying the optimal septal-coronary phenotype suitable for the uncomplicated and ultimately effective relief of LV obstruction with ASA. Owing to an uneventful long-term course, ASA, when guided by MC-3DE, may be proposed as an alternative to isolated SM for the treatment of SAM-related LV obstruction, whatever the surgical risk. However, the low applicability rate may limit the impact of ASA in an HCM patient population eligible for isolated SM. Further studies are needed to prospectively analyze the impact of MC-3DE refinement to tailor safe and long-term effective ASA in a large population with HCM at low-operative risk SM.

## Figures and Tables

**Table 1:** Characteristics of 52 patients with hypertrophic cardiomyopathy.

Patients (n°)	52
Age (years)	55 ± 15
Female (n°)	27 (52%)
Male (n°)	25 (48%)
HCM family history (n°)	8 (15%)
NYHA class III or IV dyspnea (n°)	38 (73%)
CCS class III or IV angina (n°)	18 (35%)
Syncope (n°)	12 (23%)
Smoking (n°)	11 (21%)
Atrial Fibrillation (n°)	14 (27%)
NSVT (n°)	1 (2%)
Mild Arterial Hypertension (n°)	14 (27%)
Diabetes mellitus (n°)	7 (13.5%)
Non-significant CAD (n°)	4 (8%)
Left Bundle Branch Block (n°)	3 (0.5%)
Right Bundle Branch Block (n°)	1(0.2%)
Pacemaker (n°)	1 (0.5%)
ICD (n°)	2 (0.4%)
**Medical therapy**	
Beta-blockers (n°)	50 (96%)
Calcium-antagonist (n°)	20 (38%)
Antiarrhythmic drugs (n°)	7 (13.5%)
Disopyramide (n°)	8 (15%)
**Echocardiographic parameters**	
Ejection Fraction (%)	61.58% ± 4.1
LV end-diastolic diameter (mm)	44.6 ± 4.6
Myocardial Hypertrophy	
-*Asymmetric (n°)*	47(90%)
-*Symmetric (n*°)	5 (10%)
SAM-aortic valve distance (mm)	24 ± 4
Septal thickness of target septal zone (mm)	20 ± 5
LVOT gradient (mmHg)	69.15 ± 42
SAM	
*-at rest fasting (n°)*	35 (67%)
*-at rest eating (n°)*	52 (100%)
*-inducible (n°)*	15 (29%)
SAM-related MR >2 + (n°)	42 (89%)
Mitral annular calcification (n°)	12 (23%)
Left atrium dilation (vol > 50 mL/m^2^) (n°)	14 (27%)
sPAP ≥ 50 mm Hg (n°)	14 (27%)

**NSVT**: nonsustained ventricular tachycardia; **CAD**: coronary artery disease; **ICD**: implantable cardiac defibrillator; **LV**: left ventricular; **SAM**: systolic anterior motion of mitral valve; **LVOT**: left ventricular outflow tract; **MR**: mitral regurgitation; **sPAP**: systolic pulmonary artery pressure. All patients were informed of the ASA and SM options and were aware that ultimate septal reduction modality would have required further discussion only following intracoronary myocardial contrastography results. All patients provided informed written consent for all diagnostic and interventional procedures. The study was in compliance with the Declaration of Helsinki, and the internal ethical committee approved the clinical protocol.

**Table 2:** Outcome of 15 patients with HCM undergoing alcohol septal ablation.

Variables	Baseline (15 pts)	Post-ASA (15 pts)	Pre-Discharge (15 pts)	1-Year FU (14 pts)	5-Year FU (14 pts)	10-Year FU (13 pts) *	>10-Year FU (10 pts) **
**TSZ Thickness (mm)**	20 ± 5 ^#^	19 ± 4	18 ± 7	16 ± 6	15 ± 8	15 ± 6	14 ± 2
**IVG (mm Hg)**	72 ± 44 ^##^	20 ± 3	20 ± 4	20 ± 6	18 ± 7	16 ± 7	15 ± 6
**SAM-related MR >2+** **<2+**	15 0	0 8	0 7	0 6	0 5	0 5	0 10
**LVOT-area (cm^2^)**	2.7 ± 4 ^§^	3.2 ± 2	3.3 ± 4	5.2 ± 3	6 ± 3	6 ± 4	5 ± 4
**NYHA class III–IV** **I–II**	13 2	NA NA	1 14	0 14	0 14	0 13	0 10
**Bundle Branch Block Pacemaker**	0 0	0 0	0 0	0 0	0 0	0 0	0 1
**ICD**	1	0	0	1	0	0	0
**Complex ventricular arrhythmias**	0	0	0	0	0	0	0
**ICD discharge**	0	0	0	0	0	0	0
**Medication dose reduction**	0	0	0	11	10	9	10
**Cardiac death**	0	0	0	0	0	0	0
**Noncardiac death**	0	0	0	1	0	0	0

**TSZ**: target septal zone; **IVG**: intraventricular gradient; **SAM**: systolic anterior motion of mitral valve; **MR**: mitral valve regurgitation; **LVOT**: left ventricular outflow tract; **ICD**: implantable cardiac defibrillator; **FU**: follow-up; * one patient lost at FU ** two patients lost at FU; ^#^, ^##^, ^§^*p* < 0.001 vs. 1-, 5-, 10-, >10-year FU.

## Data Availability

The data presented in this study are available on request from the corresponding author.
